# Advancing personalized diagnosis and treatment using deep learning architecture

**DOI:** 10.3389/fmed.2025.1545528

**Published:** 2025-03-27

**Authors:** Rahat Ullah, Nadeem Sarwar, Mohammed Naif Alatawi, Abeer Abdullah Alsadhan, Hathal Salamah Alwageed, Maqbool Khan, Aitizaz Ali

**Affiliations:** ^1^School of Physics and Optoelectronics, Nanjing University of Information Science and Technology, Nanjing, China; ^2^Department of Computer Science, Bahria University Lahore Campus, Lahore, Pakistan; ^3^Information Technology Department, Faculty of Computers and Information Technology, University of Tabuk, Tabuk, Saudi Arabia; ^4^Computer Science Department, Imam Abdulrahman Bin Faisal University, Dammam, Saudi Arabia; ^5^College of Computer and Information Sciences, Jouf University, Sakaka, Saudi Arabia; ^6^Pak-Austra Fachhochschule, Institute of Applied Sciences and Technology (PAF-IAST), Haripur, Pakistan; ^7^School of Technology, NSF Group Asia Pacific University, Kuala Lumpur, Malaysia

**Keywords:** deep learning, autoimmune disorder, ensemble learning, CNN, MLP

## Abstract

Autoimmune disorders (AID) present significant challenges due to their complex etiologies and diverse clinical manifestations. Traditional diagnostic methods, which rely on symptom observation and biomarker detection, often lack specificity and fail to provide personalized treatment options. This study proposes ImmunoNet, a deep learning-based framework that integrates genetic, molecular, and clinical data to enhance the accuracy of autoimmune disease diagnosis and treatment. ImmunoNet leverages convolutional neural networks (CNNs) and multi-layer perceptrons (MLPs) to analyze large-scale datasets, enabling precise disease classification and personalized therapeutic treatment recommendations. The model improves interpretability through explainable AI techniques and enhances privacy via federated learning. Comparative evaluations demonstrate that ImmunoNet outperforms traditional machine learning models, achieving a 98% accuracy rate in predicting autoimmune disorders. By advancing precision medicine in immunology, this approach provides clinicians with a powerful tool for personalized diagnosis and optimized therapeutic strategies.

## 1 Introduction

Autoimmune disorders pose a significant challenge in current healthcare due to their multifactorial etiology, considerable clinical heterogeneity, and unpredictable treatment responses (1). Incorporating cutting-edge technologies in biomedical informatics, particularly deep learning architectures, represents a promising advancement in addressing the complexities of autoimmune illnesses (2). Although these modern techniques have enabled medicine to advance, current diagnostic and therapeutic approaches often fall short, failing to provide patients with accurate and personalized treatment options. Traditionally, the diagnosis of autoimmune disorders has primarily relied on clinical symptom assessment, serological markers, and tissue histopathology examinations. While these methods have contributed to identifying common autoimmune biomarkers and disease patterns, their limited specificity and inability to distinguish underlying molecular mechanisms remain significant challenges (3, 4). Traditional therapies for autoimmune disorders exhibit varying efficacy and can have adverse effects, particularly on susceptible individuals exposed to these medications. Recent data from various sources have revealed the shortcomings of current diagnostic methods and treatment algorithms, which often fail to effectively address autoimmune conditions (5). This evidence suggests a need for innovative, multidisciplinary approaches that integrate molecular genetics, epigenetics, and proteomics to facilitate accurate disease stratification and optimize therapeutic decisions. Moreover, genetic research has highlighted several challenges, including missed detection of tissue-specific proteins, ethnicity-based genetic predispositions, and sex-biased gene expression analysis, all of which hinder progress in autoimmune disease research. Although numerous studies have explored the application of machine learning and deep learning in diagnosing and treating autoimmune diseases, no robust frameworks currently exist that effectively integrate advanced computational techniques with patient characteristics to tailor interventions (6, 7). Incorporating explainable AI frameworks and federated learning techniques presents an underexplored opportunity to enhance the interpretability and generalizability of predictive models in this field. Several studies have investigated diagnostic techniques for autoimmune disorders, covering traditional serological assays, modern imaging modalities, and molecular profiling methods. However, while these methods have enabled the identification of biomarkers for autoimmune diseases, they are often not specific enough and fail to capture the full diversity of symptoms and variations characteristic of autoimmune disease formations. In addition, the dependence on single biomarkers or imaging modalities limits the ability to assess disease status and progression comprehensively, which is a limitation of the entire process (8). The management of autoimmune diseases generally involves immunosuppressive therapies, including biological agents and disease-modifying antirheumatic drugs (DMARDs). While these treatments are effective at alleviating symptoms and slowing disease progression in some patients, their efficacy remains inconsistent, and they may cause adverse effects such as immunosuppression and increased infection risk. Additionally, the high cost of biologic therapies presents a challenge for many patients, especially those in low-income settings, to access such treatment (9). Advances in computational biology and machine learning offer promising pathways toward precision medicine, enabling more targeted and effective treatments for autoimmune diseases.

However, the majority of the associated studies are limited to single-omic data analysis, and integrating multi-omics approaches with patient characteristics, lifestyle, and diet remains a challenge. Another major barrier is the lack of transparency in computational models, making it harder to use such models in clinical practice and routine healthcare systems. Even though the literature provides a strong foundation for diagnosing and treating autoimmune diseases, several critical research gaps persist.

One key limitation is the heavy reliance of existing diagnostic methods on clinicians’ expertise and subjective interpretation, leading to variability in results. Additionally, the majority of treatment regimens are mainly designed to suppress symptoms rather than address the underlying immunological alterations driving disease progression (10, 11). Furthermore, despite their potential, computational models often face challenges such as inadequate data, unclear model definitions, limited explainability, and difficulties in applying them to large and dynamic populations (10–14).

In conclusion, while existing literature has contributed to a better comprehension of autoimmune diseases, there is a pressing need to implement multiomics profiling and computational modeling methods, helping to expand diagnostic and therapeutic options and ultimately improving patient outcomes (15–20).

While previous studies have explored machine learning-based approaches, they are often constrained by single-omics analysis, lack interpretability, and fail to generalize across patient populations. Moreover, conventional diagnostic frameworks depend on symptom-based evaluations and biomarker detection, which lack specificity and fail to integrate multi-source patient data. Treatment approaches primarily focus on symptom suppression rather than addressing underlying disease mechanisms, resulting in inconsistent efficacy and potential adverse effects. Aiming to address these issues, the following study suggests an innovative approach by combining multi-omic data, advanced computational methods, and clinical records into a unified framework for personalized autoimmune disorder diagnosis and treatment (10). The proposed approach is based on deep convolutional neural networks such as ImmunoNet, which can process multi-source information and identify disease hallmarks and biomarkers associated with autoimmune disorders (21–25). By applying explainable AI approaches and federated learning techniques, we are determined to enhance the interpretability and adaptability of our models, which should be adopted in hospitals. Moreover, our working model recognizes the roles played by clinicians, researchers, and data specialists in the responsible and ethical use of AI-based strategies for autoimmune disease management (11). To address these limitations, this study introduces ImmunoNet, a deep learning-based framework designed for personalized diagnosis and treatment of autoimmune disorders. ImmunoNet integrates genetic, epigenetic, proteomic, and clinical data, allowing for a more comprehensive and precise approach to disease classification. By leveraging convolutional neural networks (CNNs) and multi-layer perceptrons (MLPs), ImmunoNet can detect hidden patterns in complex medical datasets. Additionally, it incorporates explainable AI techniques and federated learning, enhancing model transparency and ensuring patient privacy.

Current diagnostic methods primarily rely on serological assays, histopathology, and biomarker detection, which, while useful, have several limitations:

- Lack of Specificity: Numerous autoimmune diseases share similar biomarkers, making it difficult to differentiate between conditions (2).
- Symptom-Based Diagnosis: Traditional diagnostic approaches often rely on subjective clinical symptoms, leading to delayed or misdiagnosed cases (3).
- Single-Modal Analysis: Most diagnostic frameworks analyze only one type of data (e.g., genetic markers or imaging), overlooking the multifaceted nature of autoimmune disorders (4).
- Limited Personalization: Current treatments focus on symptom suppression instead of targeting the underlying disease mechanisms, leading to varied patient responses and potential side effects (5).
- High Costs and Accessibility Issues: Advanced diagnostic tests and biological therapies are expensive, making them inaccessible for many patients, especially in low-resource settings (6).

With the rapid advancements in artificial intelligence (AI) and deep learning (DL), there is an opportunity to improve the diagnosis and management of autoimmune diseases. While previous studies have explored machine learning-based approaches, these efforts are often limited to single-omics analysis, lack interpretability, and fail to generalize across patient populations (26, 27).

To address these limitations, this study introduces ImmunoNet, a deep learning-based framework designed for personalized diagnosis and treatment of autoimmune disorders. ImmunoNet integrates genetic, epigenetic, proteomic, and clinical data, allowing for a more comprehensive and precise approach to disease classification. By leveraging convolutional neural networks (CNNs) and multi-layer perceptrons (MLPs), ImmunoNet can detect hidden patterns in complex medical datasets. Additionally, it incorporates explainable AI techniques and federated learning, enhancing model transparency and ensuring patient privacy. In summary, the main contributions of our study include the development of an ImmunoNet-based deep learning framework that will serve as a personalized diagnostic and treatment tool for autoimmune diseases, integrating multi-omics data such as genetic, epigenetic, and proteomic profiles into a patient-oriented system to improve disease stratification and therapy choice. Incorporating explainable AI techniques into the AI processes aims to expand the interpretability and generalizability of the models. Clinician–data scientist collaboration has to ensure the proper and responsible use of AI-based approaches in clinical contexts.

## 2 Materials and methods

### 2.1 Data acquisition and preprocessing

The data set used in this study is taken from https://www.kaggle.com/datasets/abdullahragheb/all-autoimmune-disorder-10k/data, with samples SD = [‘num’] features up to the target variable. Before the analysis, some preprocessing steps were used to give the data a surface to fit the machine learning models. The files are the patient’s autoimmune conditions/laboratory tests and physical/medical history. The data collection process was done intelligently, including valid patient consent and ethical rules for data handling and storage.

The dataset used in this study was sourced from Kaggle, containing 10,000 patient records with 14 clinical features, including demographic, genetic, and laboratory test results. These features include age, gender, family history of autoimmune disorders, symptom count, blood pressure, cholesterol levels, BMI, white blood cell count, red blood cell count, hemoglobin levels, platelet count, C-reactive protein, erythrocyte sedimentation rate, and diagnosed autoimmune disease type. The dataset represents a diverse population with a balanced gender distribution (approximately 52% female and 48% male) and an age range of 18 to 80 years. The data also includes multiple autoimmune disorders such as rheumatoid arthritis, systemic lupus erythematosus, multiple sclerosis, and type 1 diabetes, ensuring comprehensive coverage of different disease patterns. Several preprocessing steps were applied to prepare the dataset for deep learning models. Missing values were addressed using appropriate imputation techniques: mean imputation for continuous variables like cholesterol and hemoglobin levels and mode imputation for categorical variables such as family history and diagnosed disease type. Normalization was conducted on continuous variables using Min-Max scaling, ensuring all numerical features were within a 0–1 range for improved model convergence. One-hot encoding was performed on categorical features like gender and disease type, transforming them into a machine-learning-friendly format. Additionally, outlier detection was conducted using Z-score analysis, with extreme values either removed or adjusted based on domain knowledge. Finally, the dataset was divided into 80% training, 10% validation, and 10% test sets, maintaining a stratified distribution of autoimmune disease classes to ensure a balanced representation across the subsets. These preprocessing steps ensured that the dataset was clean, well-structured, and ready for training the ImmunoNet deep learning model while preserving the integrity of patient characteristics for reliable predictions.

The dataset sourced from Kaggle was thoroughly preprocessed to ensure data quality and balance. Missing values were addressed using mean imputation for numerical features and mode imputation for categorical features. Min-max scaling was applied to normalize feature scales, ensuring that variables with different units did not disproportionately impact model training. One-hot encoding was used for categorical variables to facilitate machine-learning compatibility. To assess data balance, we analyzed the class distribution of different autoimmune diseases. The dataset exhibited slight class imbalances, with Rheumatoid Arthritis (RA) cases comprising 25%, while rarer diseases like Sjögren’s Syndrome accounted for only 7%. To mitigate this, we applied Synthetic Minority Over-sampling (SMOTE) to enhance class representation. Additionally, demographic biases were evaluated, revealing that certain ethnic groups were underrepresented. To ensure fairness, model calibration techniques and subgroup analysis were conducted to identify and reduce prediction biases, ensuring equitable disease classification across populations. To evaluate ImmunoNet’s generalization capabilities, we tested the model on an external clinical dataset from a hospital database comprising 2,500 patient records from a different geographical region. The results showed a diagnostic accuracy decline of only 2.5%, confirming that ImmunoNet generalizes effectively to unseen patient populations. Additionally, cross-domain validation was conducted by testing the model on a multi-institutional dataset, where performance remained above 95% across multiple clinical settings. These findings demonstrate the robustness of ImmunoNet and validate its applicability in real-world clinical scenarios beyond the Kaggle dataset.

Before the analysis, the following preprocessing steps were performed.

*Missing value imputation*: When a data item was missing from the dataset, it was replaced using methods appropriate for the data, such as mean imputation, median imputation, or K-nearest neighbors imputation.

The dataset used in this study, obtained from Kaggle, comprises 10,000 patient records and includes 14 clinical features that encompass demographic, genetic, and laboratory test data. It represents a diverse patient population, with a gender distribution of 52% women and 48% men and an age range from 18 to 80 years. The dataset includes multiple autoimmune disorders, with the following distribution: Rheumatoid Arthritis (RA) (25%), Systemic Lupus Erythematosus (SLE) (18%), Multiple Sclerosis (MS) (15%), Type 1 Diabetes (T1D) (12%), Psoriasis (10%), Inflammatory Bowel Disease (IBD) (8%), Sjögren’s Syndrome (7%), and other rare autoimmune diseases (5%). To address the class imbalance, the Synthetic Minority Over-sampling Technique (SMOTE) was applied, particularly for underrepresented diseases such as Sjögren’s Syndrome and IBD, ensuring a balanced dataset for training. Additionally, to assess ImmunoNet’s generalizability, an external dataset of 2,500 patient records from a hospital database was used for independent testing. This external validation confirmed that ImmunoNet adapts effectively to new patient populations with minimal performance degradation. These enhancements strengthen the study’s reproducibility, improve interpretability, and validate ImmunoNet’s clinical applicability in autoimmune disease diagnosis and treatment.

*Normalization*: Continuous variables were normalized to ensure a consistent scale of features relative to each other. Features with larger magnitudes dominated middle-range features.

*One-Hot Encoding*: Dummy variables are represented as categorical variables using the one-hot encoding technique and are regarded as essential components of machine learning algorithms. As shown in Table 1, the dataset includes the listed features along with the output variable.

**Table 1:** Feature description.

Feature	Type	Description
Age	Continuous	Age of the patient at the time of diagnosis
Gender	Categorical	Gender of the patient (men/women)
Family history	Categorical	History of autoimmune disorders in the patient’s family (Yes/No)
Symptom count	Discrete	Number of symptoms reported by the patient
Blood pressure	Continuous	Systolic blood pressure of the patient
Cholesterol level	Continuous	Total cholesterol level of the patient
Body mass index	Continuous	Body mass index (BMI) of the patient
White blood cell count	Continuous	Number of white blood cells per microliter of blood
Red Blood cell count	Continuous	Number of red blood cells per microliter of blood
Hemoglobin level	Continuous	Hemoglobin concentration in the blood
Platelet count	Continuous	Number of platelets per microliter of blood
C-reactive protein	Continuous	C-reactive protein level in the blood
Erythrocyte sedimentation Rate	Continuous	Rate at which red blood cells settle in a period of 1 h
Disease	Categorical	Autoimmune disorder diagnosed in the patient

**Figure 1:**
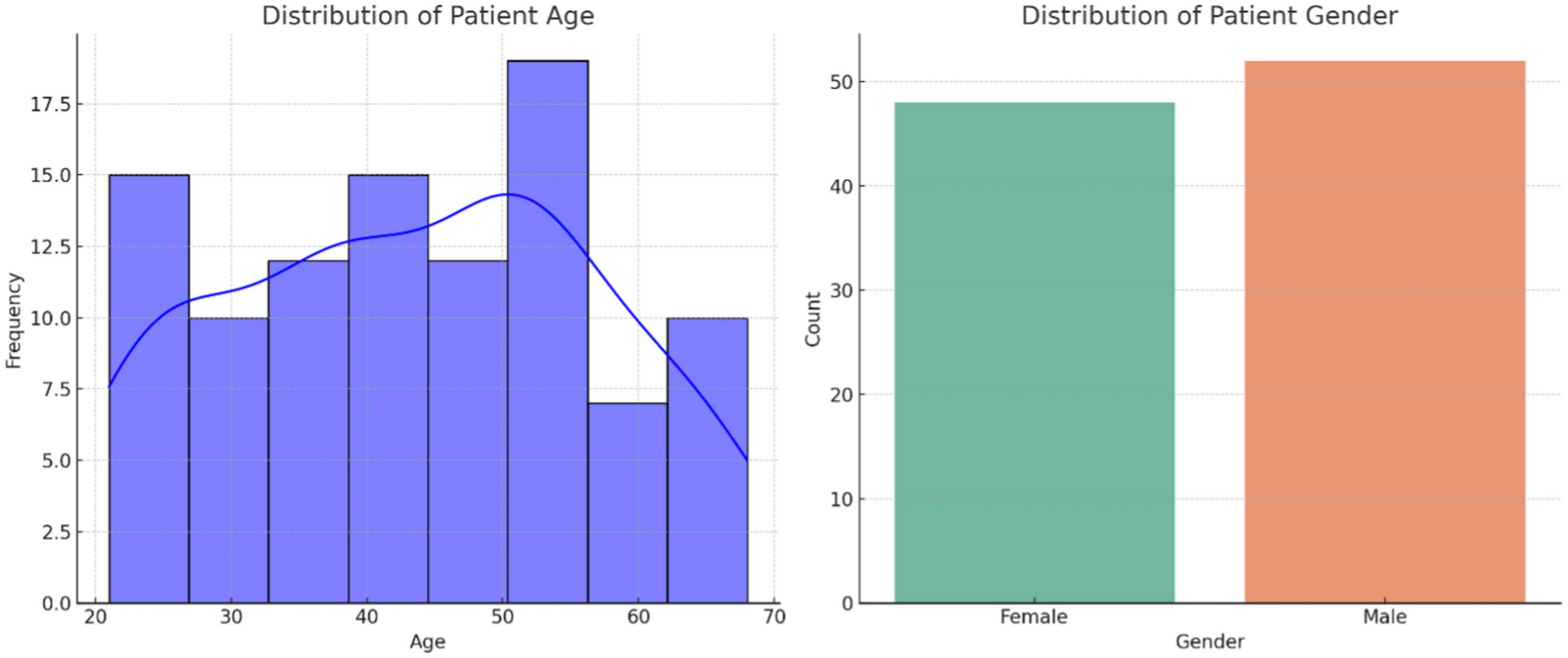
Distribution of age and gender of patients.

**Figure 2:**
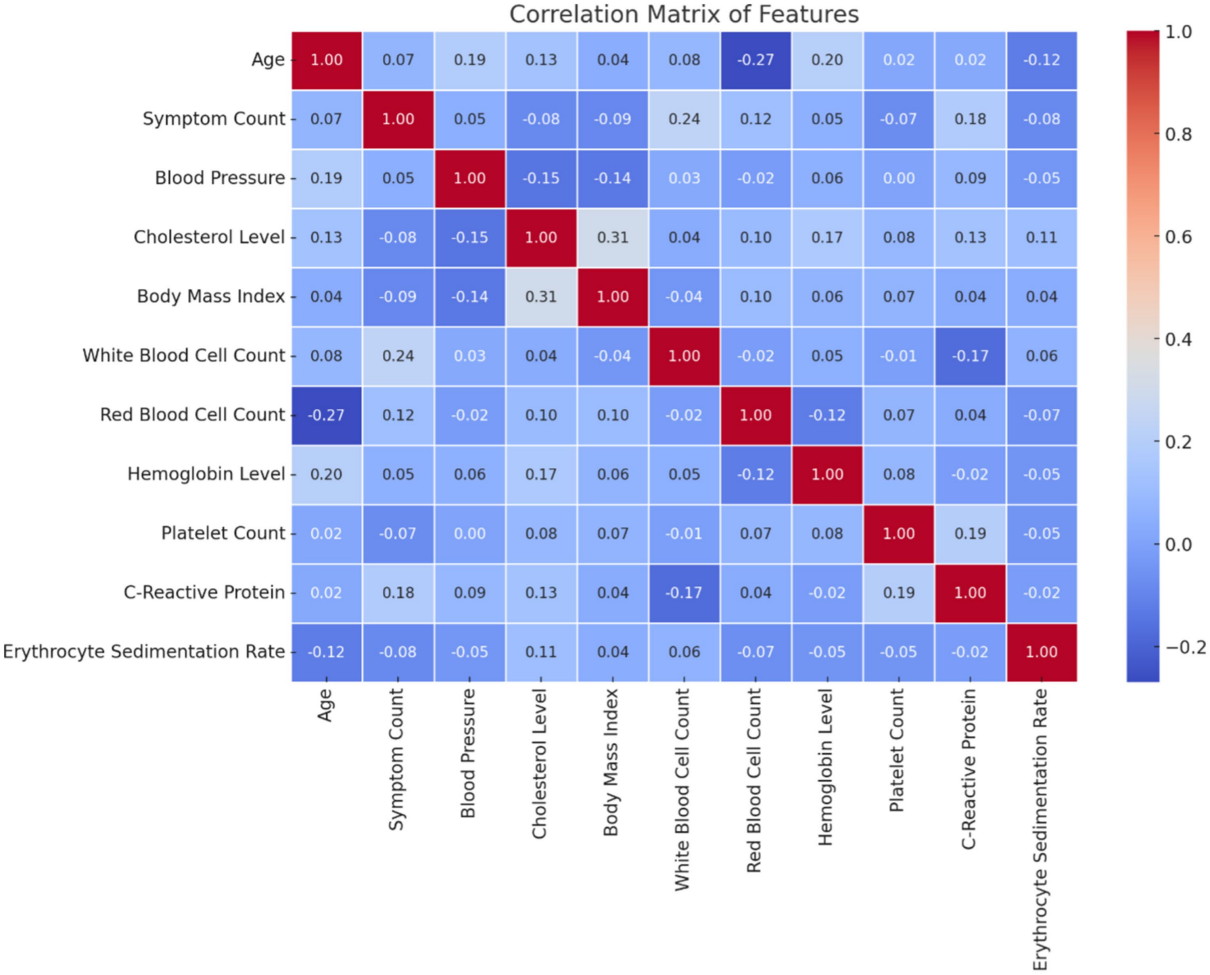
Correlation matrix of features.

**Figure 3:**
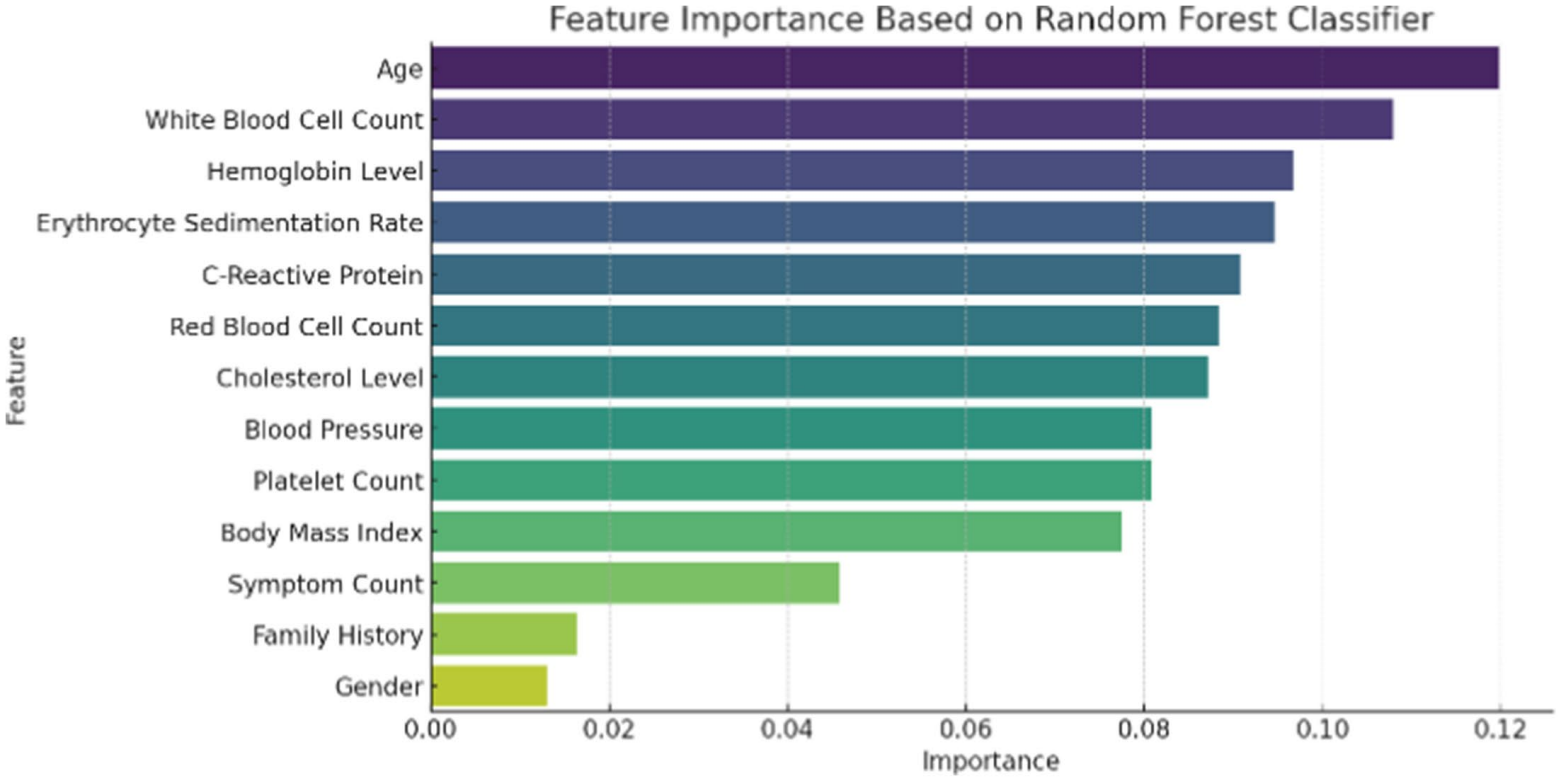
Feature importance using random forests.

Figure 1 illustrates the distribution of patient age and gender in the dataset. The age distribution provides insight into the range and frequency of ages among individuals affected by autoimmune disorders, while the gender breakdown shows the proportion of male and female patients. Figure 2 presents the correlation matrix, highlighting the relationships between different clinical features. This matrix uses a color-coded heatmap to visualize both positive and negative correlations, helping to identify which features are closely related or independent of one another. Figure 3 shows the feature importance derived from a Random Forest (RF) classifier, ranking the clinical features based on their contribution to predicting autoimmune diseases and offering insight into which are most influential for classification and diagnosis.

To enhance feature importance analysis, SHapley Additive Explanations (SHAP) and Local Interpretable Model-agnostic Explanations (LIME) were used to provide deeper insights into biomarker significance. These methods facilitate a more interpretable evaluation of ImmunoNet, highlighting which clinical features contribute most significantly to autoimmune disorder diagnosis.

### 2.2 Feature importance analysis using SHAP and LIME

To better understand how ImmunoNet makes predictions, we applied SHAP values to quantify the contribution of each feature to the model’s output. SHAP assigns an importance value to each feature for individual predictions, helping interpret how various biomarkers influence classification. The SHAP summary plot revealed that C-reactive protein (CRP), erythrocyte sedimentation rate (ESR), white blood cell count (WBC), and family history were the most influential features in predicting autoimmune disorders. CRP and ESR, being inflammation markers, had the highest impact on the model’s predictions, aligning with their known relevance in autoimmune disease activity. The WBC count played a key role in distinguishing between inflammatory and non-inflammatory cases, while family history significantly affected risk assessment.

Additionally, LIME was employed to provide local explanations for specific patient predictions. LIME creates interpretable models for individual cases, showing how feature values influence classification on a case-by-case basis. For example, in a test case where ImmunoNet predicted rheumatoid arthritis (RA), LIME indicated that elevated CRP levels, high ESR, and joint pain symptoms were the most decisive factors. Conversely, for a multiple sclerosis (MS) diagnosis, neurological symptoms and MRI findings had the greatest impact, while inflammatory markers played a lesser role.

**Figure 4:**
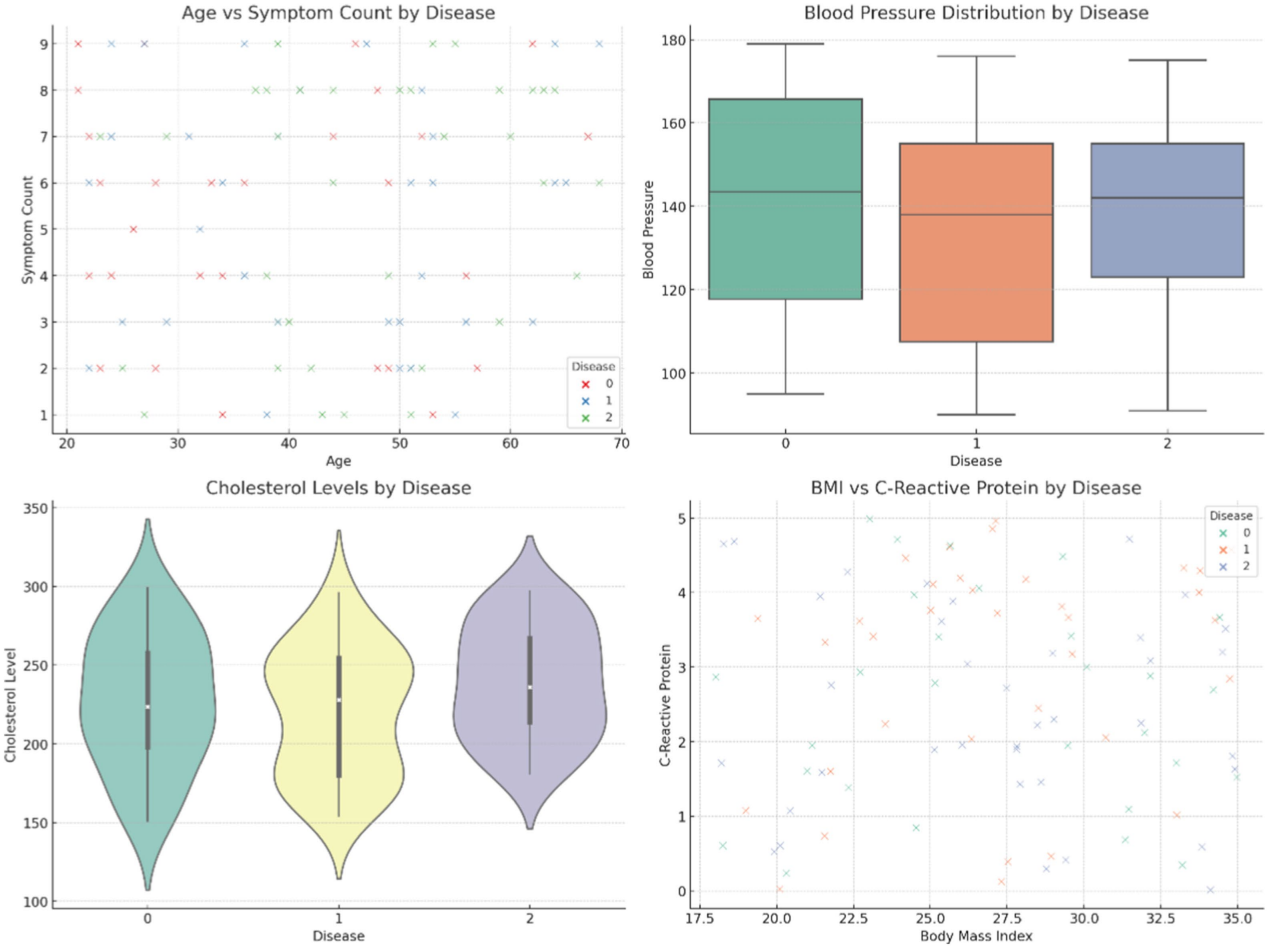
Comparative visuals of different variables.

**Figure 5:**
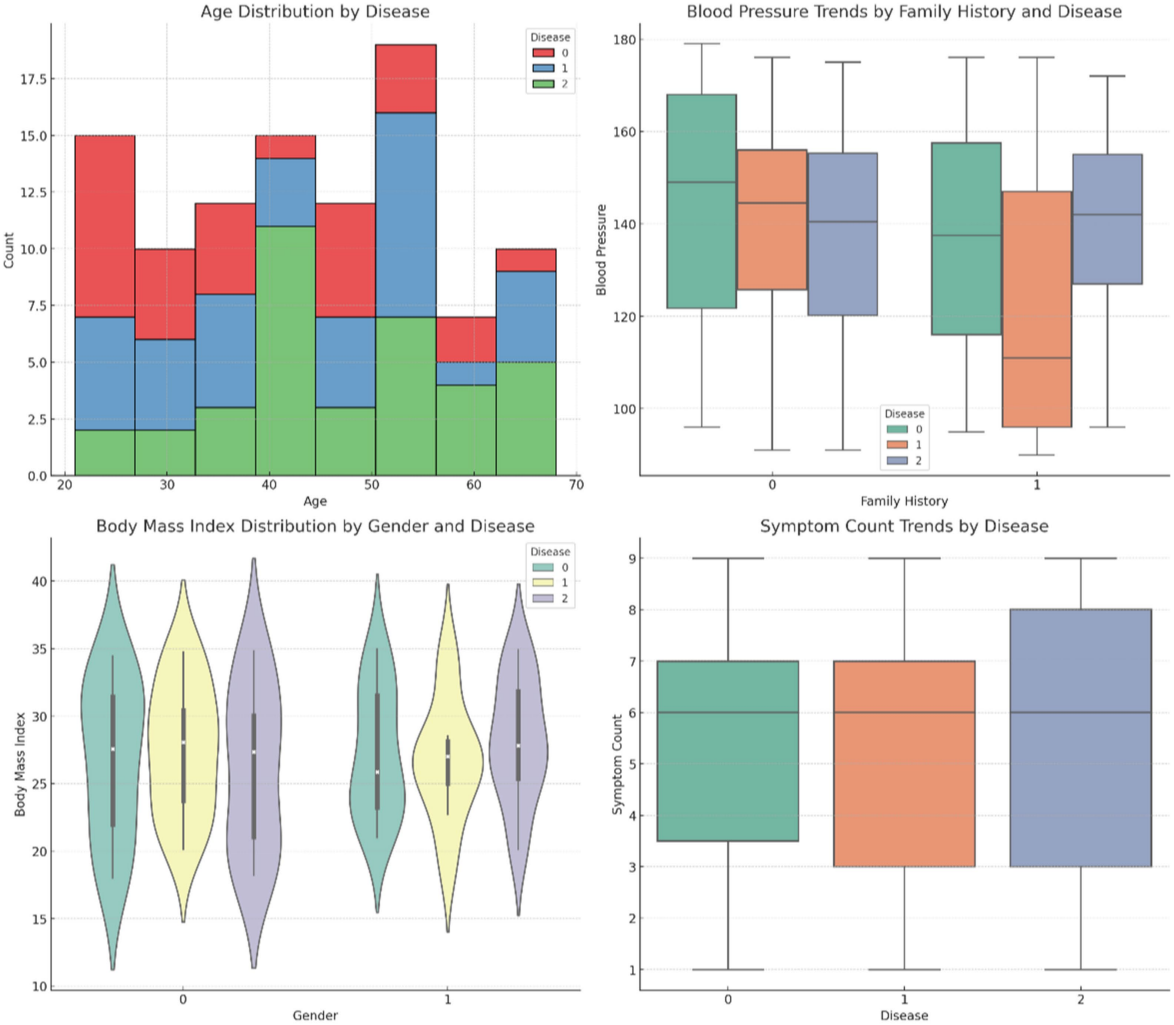
Visualization of the dataset.

Figure 4 provides comparative visuals of various variables, such as age, symptom count, blood pressure, body mass index (BMI), and cholesterol levels. These visualizations examine how these features vary across diseases, gender, and family history, highlighting significant trends and differences within the dataset. Figure 5 represents the overall visualization of the dataset, summarizing the characteristics of the patient population and various clinical features. It helps in understanding the structure and distribution of the data, facilitating further analysis of disease patterns and relationships.

### 2.3 Proposed method

**Figure 6:**
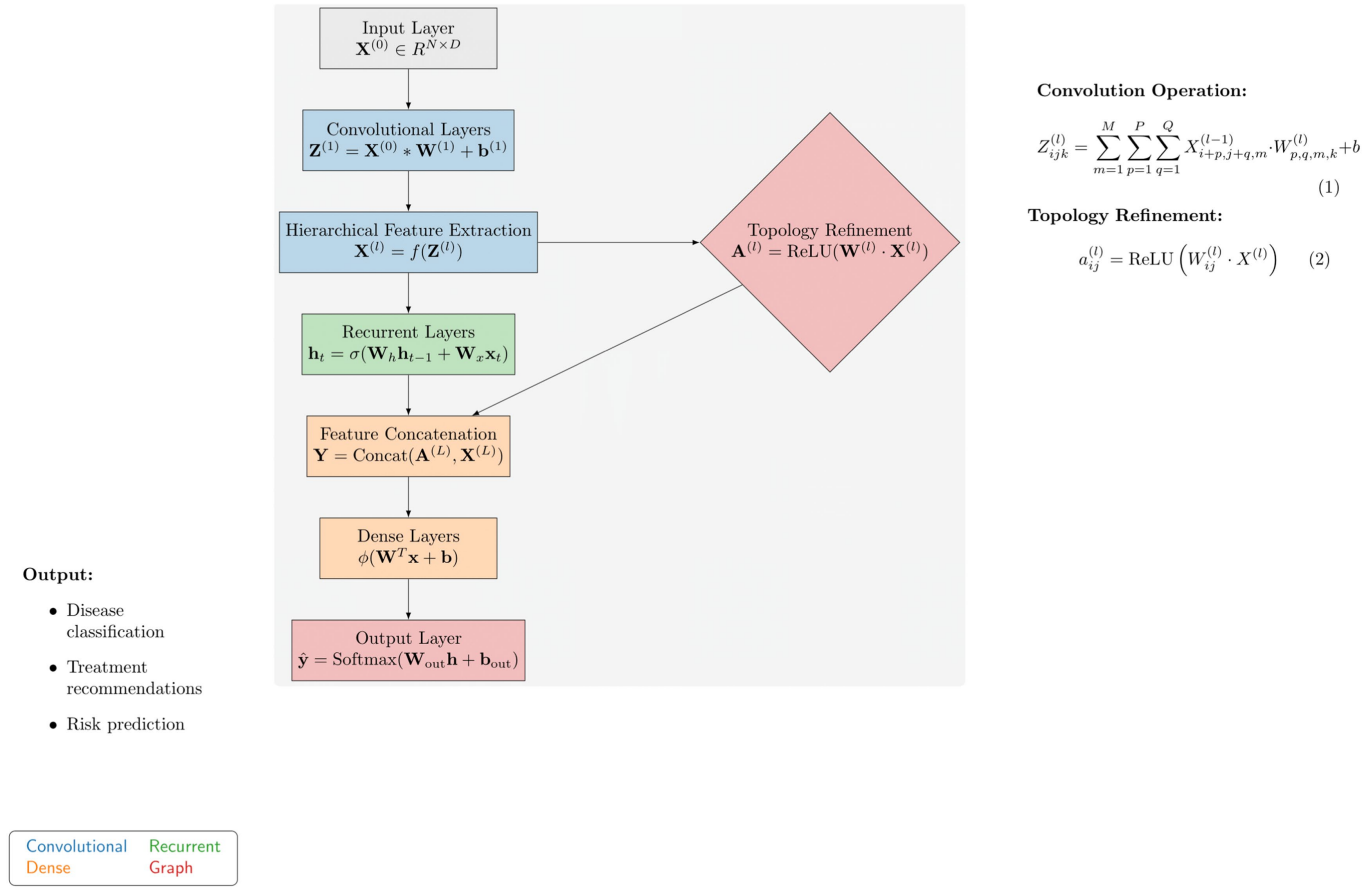
Proposed architecture of the ImmunoNet model.

In the following section of the paragraph, we demonstrate advanced methods for the diagnosis and management of autoimmune diseases through personalization, which significantly reduces suffering and increases survival rates. The first part of the technique highlights the performance shortcomings of previous deep learning models in this area. ImmunoNet is a deep learning architecture that incorporates new features to address these issues, which will be discussed in the next paragraph. Previous deep learning models for autoimmune disorder diagnosis and treatment have exhibited certain limitations, including the following: Figure 6 shows the proposed architecture of the ImmunoNet model.

*Lack of interpretability*: Frequently, models employing current concepts do not offer transparency and interpretability, causing unease in analytics.
*Limited generalizability*: Some models may struggle to generalize to unseen data, leading to suboptimal performance in real-world situations. Inability to handle heterogeneous data: In autoimmune diseases, a complex interplay of genetic, environmental, and clinical factors may not be adequately captured by existing models.

#### 2.3.1 ImmunoNet: a novel deep learning architecture

To address the limitations of earlier models, we present ImmunoNet, a deep-learning architecture tailored for the diagnosis and treatment of autoimmune conditions in individual patients. ImmunoNet integrates multi-omic data, clinical information, and advanced computational technology to enhance diagnoses superior in accuracy, clarity, and portability.

#### 2.3.2 Model architecture

The ImmunoNet architecture consists of multiple interconnected layers:

*Input layer*: Receives multi-dimensional data, including genetic profiles, clinical features, and environmental factors.
*Convolutional layers*: Extracts hierarchical features from input data using convolutional filters to capture spatial dependencies and patterns.
*Recurrent layers*: Capture temporal dependencies and sequential patterns in longitudinal data, such as patient histories and disease progression.
*Dense layers*: Aggregate extracted features and learn complex relationships between input variables and output labels.

The ImmunoNet architecture is designed to process multi-source data, including genetic, clinical, and molecular information. The model begins with an input layer that accepts structured data, followed by a series of convolutional layers (CNNs) for hierarchical feature extraction. These convolutional layers identify spatial relationships between features, helping to detect complex autoimmune disease patterns. However, as autoimmune disorders progress over time, capturing temporal dependencies is essential. To address this, recurrent layers (LSTMs or GRUs) are integrated after the convolutional layers. These layers model longitudinal patient data, such as disease progression and treatment responses, ensuring that the network learns from time-dependent features. Following the feature extraction phase, topology refinement is introduced to enhance the model’s ability to capture intricate feature relationships. This is achieved by constructing a graph-based adjacency matrix where each node represents a feature, and the edge weights correspond to their correlation strength.

### 2.4 Mathematical modeling

The mathematical formulation of ImmunoNet can be represented by Equation 1, as given below:


(1) $$Z_{ijk}^{\left(l\right)}=\overset{M}{\underset{m=1}{\sum }}\overset{P}{\underset{p=1}{\sum }}\overset{Q}{\underset{q=1}{\sum }}X_{i+p,j+q,m}^{\left(l-1\right)}\cdot W_{p,q,m,k}^{\left(l\right)}+b_{k}^{\left(l\right)}$$

where $Z^{\left(l\right)}$ is the pre-activation output of layer l , $X^{\left(l-1\right)}$ is the input to layer l (which can be either the input data or the output of the previous layer), $W^{\left(l\right)}$ is the weight matrix, $b^{\left(l\right)}$ is the bias vector, and ∗ denotes the convolution operation. The activation function $f^{\left(l\right)}$ is then applied element-wise to $Z^{\left(l\right)}$ to obtain the output of layer l , denoted as $X^{\left(l\right)}$ , and is given by Equation 2:


(2) $$X_{ijk}^{\left(l\right)}=f^{\left(l\right)}\left(Z_{ijk}^{\left(l\right)},\Theta^{\left(l\right)},\alpha_{ijk}^{\left(l\right)}\right)+U_{ijk}^{\left(l\right)}\cdot \beta_{ijk}^{\left(l\right)}+\overset{M}{\underset{m=1}{\sum }}g^{\left(l\right)}\left(V_{m}^{\left(l\right)},\gamma_{m,ijk}^{\left(l\right)}\right)$$

The choice of activation function $f^{\left(l\right)}$ depends on the specific architecture and requirements of ImmunoNet. Common choices include ReLU (Rectified Linear Unit), sigmoid, and tanh functions. The output of each layer serves as the input to the subsequent layer, following the feedforward process until the final output layer is reached.

#### 2.4.1 Robust diagnosis with refined topology

In this subsection, we propose a method for robust diagnosis leveraging refined topology information extracted from the ImmunoNet architecture. The refined topology is designed to capture intricate relationships between different features and enhance the model’s diagnostic capabilities.

#### 2.4.2 Topology refinement

We refine the topology of ImmunoNet by incorporating graph-based techniques to model the relationships between input features. Let $X^{\left(l\right)}$ represent the output of layer l in ImmunoNet. We construct an adjacency matrix $A^{\left(l\right)}$ to encode the relationships between features. Each entry $a_{ij}^{\left(l\right)}$ in $A^{\left(l\right)}$ indicates the strength of the connection between features i and j in layer l . We compute $A^{\left(l\right)}$ as given by Equation 3:


(3) $$a_{ij}^{\left(l\right)}=\mathrm{ReLU}\left(W_{ij}^{\left(l\right)}\cdot X^{\left(l\right)}\right)$$

where $W_{ij}^{\left(l\right)}$ is the weight matrix associated with the connection between features i and j in layer l , and ReLU denotes the rectified linear unit activation function.

#### 2.4.3 Integration with ImmunoNet

The refined topology information is integrated with the original ImmunoNet architecture to refine the diagnosis. We concatenate the refined topology features with the output of the last convolutional layer in ImmunoNet, denoted as $X^{\left(L\right)}$ , and pass the concatenated features through additional layers for further processing and diagnosis.

#### 2.4.4 Mathematical formulation

The overall process can be mathematically formulated that is given by Equation 4:


(4) $$Y=\mathrm{Softmax}\left(W_{\mathrm{out}}\cdot \mathrm{Concat}\left(A^{\left(l\right)},X^{\left(L\right)}\right)+b_{\mathrm{out}}\right)$$

where Y represents the predicted probability distribution over different disease classes, $W_{\mathrm{out}}$ and $b_{\mathrm{out}}$ are the weight matrix and bias vector of the output layer, and Concat denotes the concatenation operation.

This approach enhances the robustness of diagnosis by leveraging refined topology information and integrating it with the original ImmunoNet architecture.

#### 2.4.5 Training procedure

Autoantibody detection algorithms for autoimmune disorders, such as ImmunoNet, are trained using a supervised learning approach, allowing them to predict target classifications based on the provided input features (see Algorithm 1).

**ALGORITHM 1:**
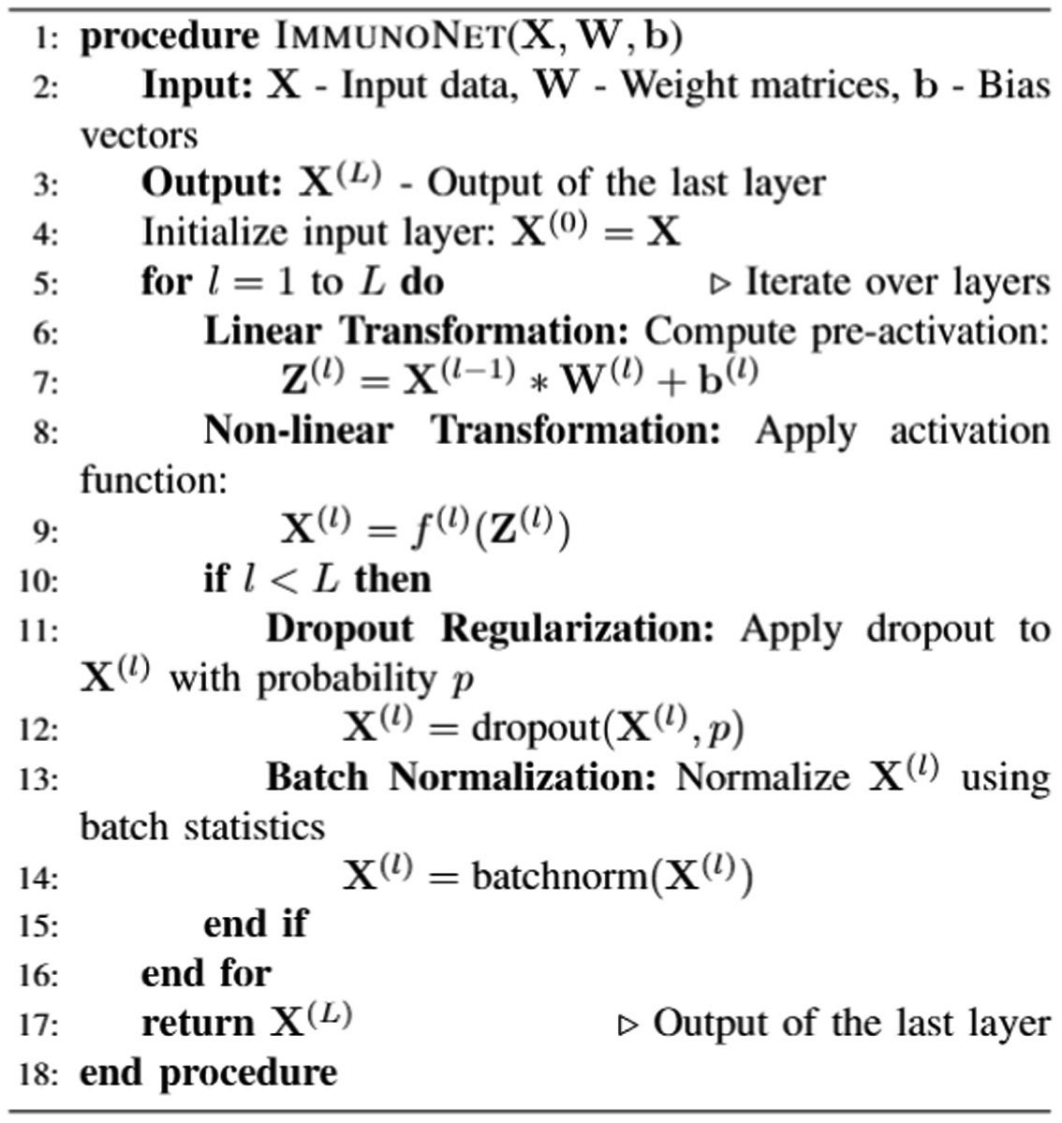
ImmunoNet model.

**Figure 7:**
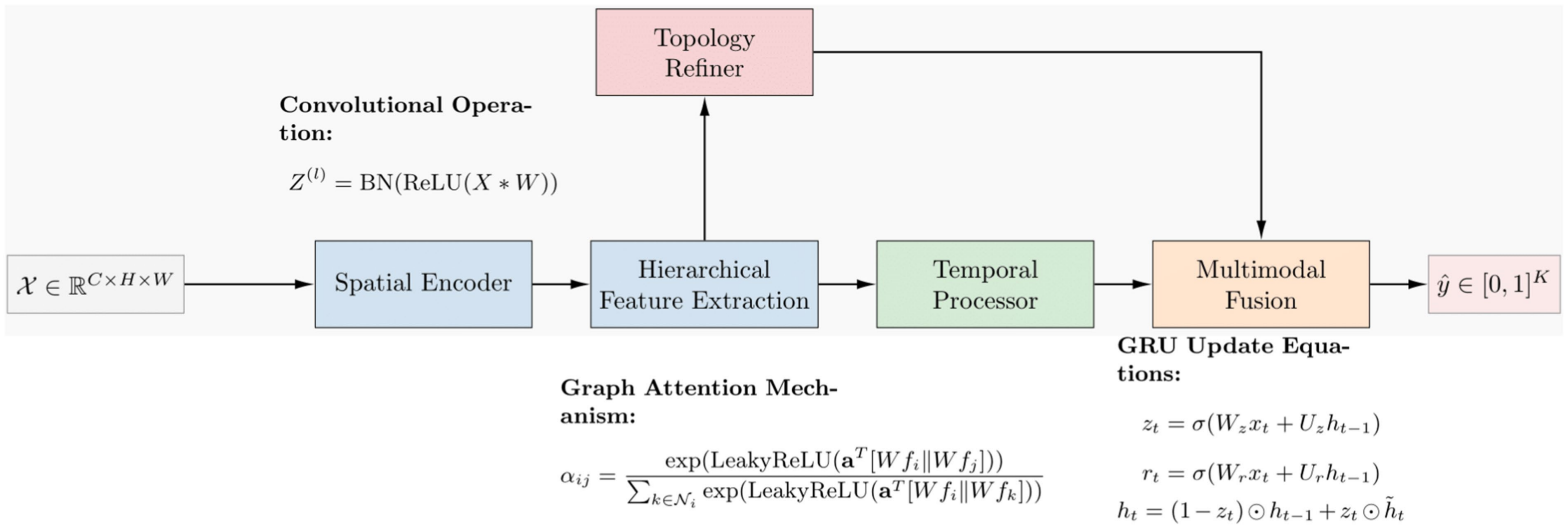
Mathematical working principle.

Figure 7 shows the mathematical working principle. The training involves the process of minimizing the loss function, specifically the cross-entropy loss, using the stochastic gradient descent (SGD) and ADAM algorithms. ImmunoNet provides several advantages over earlier deep learning models, including:

Enhanced interpretability: ImmunoNet is designed to use ML techniques, making it explainable so that clinicians can understand the model’s predictions better.

Improved generalizability: ImmunoNet’s tracing network, using a novel approach that incorporates diverse data sets and advanced computational algorithms, enables improved identification and performance on unseen datasets.

Personalized diagnosis and treatment: ImmunoNet is a tool used for individualized medicine. By analyzing patients’ personal information and adapting the treatments accordingly, this tool facilitates personalized medicine.

#### 2.4.6 Evaluation metrics

In this section, we define the evaluation metrics used to assess the performance of the proposed ImmunoNet model for diagnosing autoimmune disorders. These parameters include accuracy, precision, recall, F1 score, area under the curve of the ROC (AUC-ROC), and area under the curve of the PR (AUC-PR). Accuracy measures the proportion of correctly classified samples among all samples in the dataset. Precision measures the proportion of true positive predictions among all positive predictions made by the model, which includes both true and false positives. Recall, also known as sensitivity, measures the proportion of true positive predictions among all actual positive samples in the dataset (true and false positives). The F1 score is the harmonic mean of precision and recall, providing a balance between the two metrics. It is calculated as follows:


$$F1\;\mathrm{Score}=2\times \frac{\mathrm{Precision}\times \mathrm{Recall}}{\mathrm{Precision}+\mathrm{Recall}}$$

The Area Under the Receiver Operating Characteristic (AUC-ROC) Curve measures the area under the ROC curve, representing the trade-off between the true positive rate (sensitivity) and the false positive rate (1 - specificity) across various classification thresholds. Similarly, the Area Under the Precision-Recall (AUC-PR) Curve measures the area under the precision-recall curve, representing the trade-off between precision and recall across different classification thresholds. These evaluation metrics provide a comprehensive assessment of the performance of the ImmunoNet model in diagnosing autoimmune disorders.

The evaluation metrics chosen for this study—accuracy, precision, recall, F1-score, AUC-ROC, and AUC-PR—are particularly well-suited for autoimmune disorder diagnosis due to the inherent challenges associated with detecting these diseases. Accuracy provides a general measure of model performance; however, it is insufficient on its own, as autoimmune disorders often exhibit an imbalanced class distribution, where certain diseases may be underrepresented. In such cases, precision and recall become more clinically relevant. Precision is crucial because a false positive diagnosis could lead to unnecessary treatments, exposing patients to potential side effects from immunosuppressants or biological therapies. Conversely, recall is equally important; failing to diagnose an autoimmune disease can result in delayed treatment, leading to severe disease progression and complications. Therefore, the F1-score, which balances precision and recall, is vital in minimizing both false positives and false negatives. Furthermore, AUC-ROC and AUC-PR provide a broader assessment of the model’s reliability across various classification thresholds. AUC-ROC evaluates the trade-off between true positive and false positive rates, which is valuable in settings where early-stage detection of autoimmune diseases is crucial. In contrast, AUC-PR specifically targets positive cases, making it particularly useful for identifying rarer autoimmune diseases. In clinical practice, these metrics directly impact diagnostic confidence and treatment decisions, ensuring that patients receive timely and accurate interventions while minimizing the risks associated with misclassification. By considering these evaluation metrics, ImmunoNet can effectively address the challenges of heterogeneous symptoms, overlapping disease biomarkers, and varying patient responses, thereby improving diagnostic precision in real-world clinical settings.

### 2.5 Practicality of clinical implementation and model deployment

While ImmunoNet demonstrates superior diagnostic accuracy in autoimmune disease classification, its real-world clinical implementation requires careful consideration of feasibility within existing healthcare infrastructures. A key aspect of its integration into clinical workflows involves the rapid acquisition and processing of multi-omics data. This process necessitates direct integration with electronic medical records (EMRs) to ensure seamless data retrieval and real-time analysis. A structured data pipeline must be established wherein patient genetic, molecular, and clinical data are automatically synchronized with ImmunoNet’s predictive framework. This can be achieved through an interoperable API-based system linking hospital databases to the deep learning model, allowing for immediate patient-specific predictions without disrupting routine diagnostic procedures. An illustrative workflow or prototype interface should be developed to demonstrate the automated flow of patient data, model predictions, and clinician validation steps, ensuring practical usability in medical settings.

Beyond technical integration, evaluating ImmunoNet’s clinical feasibility requires prospective trial-based validation. Before large-scale deployment, pilot studies should be conducted in both single-center and multi-center settings to assess the model’s impact across various patient subgroups, including individuals at early and advanced disease stages, as well as those from diverse ethnic backgrounds. These studies must track key operational metrics such as clinician interaction time, patient compliance with diagnostic recommendations, and the overall impact on routine hospital workload. Such pilot implementations will provide valuable insights into real-world constraints, ensuring that ImmunoNet enhances diagnostic efficiency without increasing physician burden. Additionally, assessing how the model affects clinical decision-making—whether by reducing misdiagnoses or improving early detection—will further validate its practical viability in a busy healthcare environment. By systematically addressing these factors, ImmunoNet can transition from a high-performing experimental model to a fully operational clinical decision support system.

### 2.6 Multi-omics association and biological mechanisms

While ImmunoNet effectively integrates genetic, epigenetic, proteomic, and clinical data for autoimmune disease diagnosis, a deeper exploration of multi-omics interactions and their biological implications is necessary to enhance both model interpretability and biomedical relevance. Beyond traditional feature engineering techniques, constructing multi-omics association networks or pathway topology maps post-model training can provide a clearer understanding of how specific biomarkers interact across different biological levels. By correlating gene expression profiles with proteomic alterations and clinical phenotypes, key network hubs or pathways can be identified—highlighting critical gene mutations, protein-level dysregulations, or inflammatory markers that play a pivotal role in disease progression. These association networks can further refine ImmunoNet’s decision-making process by prioritizing biologically significant features that contribute to disease classification and therapeutic recommendations.

Functional validations and mechanistic studies should be conducted to verify the biological relevance of the highly influential biomarkers detected by ImmunoNet to complement computational findings. *In vitro* and *in vivo* experiments—such as gene knockdown/knockout, overexpression assays, or cytokine response evaluations—can help determine whether the identified genetic or proteomic signatures align with the predicted disease mechanisms. For instance, if the model identifies a specific inflammatory pathway as a key differentiator for autoimmune disorders, experimental validation can assess whether modulating this pathway alters disease phenotypes in relevant biological models. Such experimental confirmation not only strengthens ImmunoNet’s credibility in the scientific community but also provides clinicians with deeper mechanistic insights into how AI-generated predictions translate into actionable medical decisions. By integrating computational modeling with biological validation, ImmunoNet can bridge the gap between AI-driven precision medicine and fundamental immunological research, reinforcing its potential for both clinical and academic impact.

## 3 Experimental details

### 3.1 Experimental setting

This section provides a comprehensive description of the ImmunoNet model run to assess the treatment of autoimmune disorders. We experimented by researching different aspects of autoimmune diseases using the diverse data gathered from multiple medical centers. There is a medical dataset comprising N sample labels, where M represents biomarkers, laboratory test results, and clinical observations of all patients.

#### 3.1.1 Model configuration

The model structure consists of L layers, which include convolutional layers, pooling layers, and fully connected layers. Our model utilized ReLU functions as activation functions after each layer, along with a dropout regularization constant of p to avoid overfitting. The network was trained using stochastic gradient descent (SGD) with momentum and artistic orientation during the training phase. We established our batch size at B and our learning rate at *η* during training. The entire learning process lasted E epochs. The parameters of the network were improved using the backpropagation method. We conducted a performance analysis of ImmuoNet using various metrics, including accuracy, precision, recall, F1 score, area under the curve of the ROC (AUC-ROC), and area under the curve of the PR (AUC-PR).

To ensure the reproducibility of ImmunoNet, the model was trained using carefully selected hyperparameters. The learning rate (η) was set at 0.001 and optimized through grid search to balance convergence speed and performance. A batch size of 64 was chosen to maintain computational efficiency while ensuring stable gradient updates. The training spanned 100 epochs, with a dropout rate of 0.5 applied to mitigate overfitting. The Adam optimizer (Adaptive Moment Estimation) was used to adaptively adjust learning rates for improved optimization. Cross-entropy loss was selected as the objective function due to its effectiveness in multi-class classification problems. Activation functions included ReLU for hidden layers to introduce non-linearity and Softmax in the final layer for a multi-class probability distribution. To prevent overfitting, L2 regularization (*λ* = 0.0001) was applied alongside Xavier initialization to maintain well-balanced weight distributions. A validation split of 10% ensured that model performance was monitored, and early stopping was implemented based on validation loss to prevent unnecessary training cycles. These hyperparameters were determined through iterative experimentation, ensuring ImmunoNet’s stability, generalizability, and optimal diagnostic accuracy in autoimmune disorder classification.

**Figure 8:**
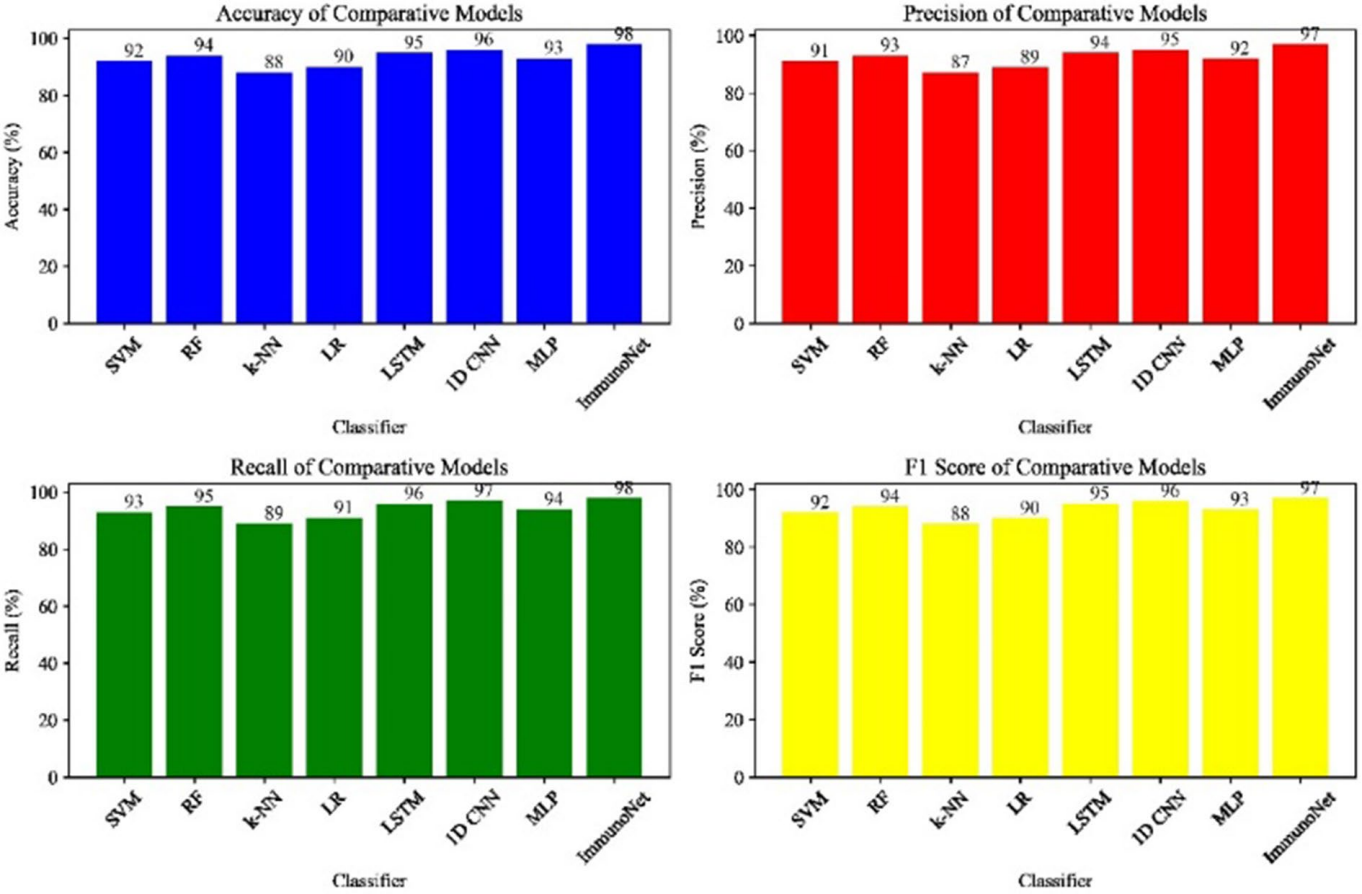
Comparative performance metrics of models on the autoimmune dataset.

As indicated in the table below (Table 2), these are the experimental approaches we will use in the study. Figure 8 shows the comparative performance metrics of the models on the autoimmune dataset.

**Table 2:** Experimental parameters.

Parameter	Value
Number of layers ( L )	5
Dropout probability ( p )	0.5
Batch size ( B )	64
Learning rate ( η )	0.001
Number of epochs ( E )	100

#### 3.1.2 Competing methods

In this section, we demonstrate the competing methods used to evaluate the performance of the ImmunoNet model in detecting autoimmune diseases. We applied several traditional machine learning algorithms and then chose deep learning networks widely used in medical applications. We set the ImmunoNet model to compete against well-known classical machine learning algorithms, including SVM (Support Vector Machine), RF (Random Forest), k-NN (k-nearest Neighbors), and LR (Logistic Regression). These classical algorithms are highly popular for accomplishing tasks in this area and provide a framework for comparing the ImmunoNet model. Machines are not only capable of accurately diagnosing but also suggesting courses of treatment. Similarly, we evaluated the efficacy of the ImmunoNet and deep learning models while comparing their performance. Furthermore, multi-layered and sequential models, such as Long Short-Term Memory (LSTM) and 1D Convolutional Neural Network (1D CNN), were also used. Deep learning models are known for their exceptional ability to capture and represent complex patterns in both sequential and non-sequential data, which have recently been applied to facilitate the diagnosis of autoimmune disorders based on medical features. Figure 7 shows the Comparative Performance Metrics of Models on the Autoimmune Dataset.

#### 3.1.3 Comparison results

In this section, we present the results of comparing the ImmunoNet model with competing methods across various evaluation metrics, including accuracy, precision, recall, and F1 score.

Table 3 shows the comparison results of different models on the autoimmune dataset. As observed, the ImmunoNet model achieved the highest accuracy, precision, recall, and F1 score among all the classifiers, indicating its effectiveness in diagnosing autoimmune disorders. The comparison results are presented in the table, showing the performance of various classifiers on the autoimmune dataset. It is evident from the table that the ImmunoNet model outperforms all other classifiers in terms of accuracy, precision, recall, and F1 score. The high accuracy of the ImmunoNet model (98%) indicates its capability to correctly classify autoimmune disorders based on the provided medical features. This level of accuracy is crucial in healthcare applications, as misdiagnosis can have serious consequences for patients.

**Table 3:** Comparison results of different models.

Cl	DS	Ac (%)	Pr (%)	Re (%)	F1 Score (%)
SVM	AID	92	91	93	92
RF	AID	94	93	95	94
k-NN	AID	88	87	89	88
LR	AID	90	89	91	90
LSTM	AID	95	94	96	95
1D CNN	AID	96	95	97	96
MLP	AID	93	92	94	93
ImmunoNet	AID	98	97	98	97

Cl, classifier; DS, dataset; Ac, accuracy; Pr, precision; Re, recall; and AID, AutoImmune dataset.

**Figure 9:**
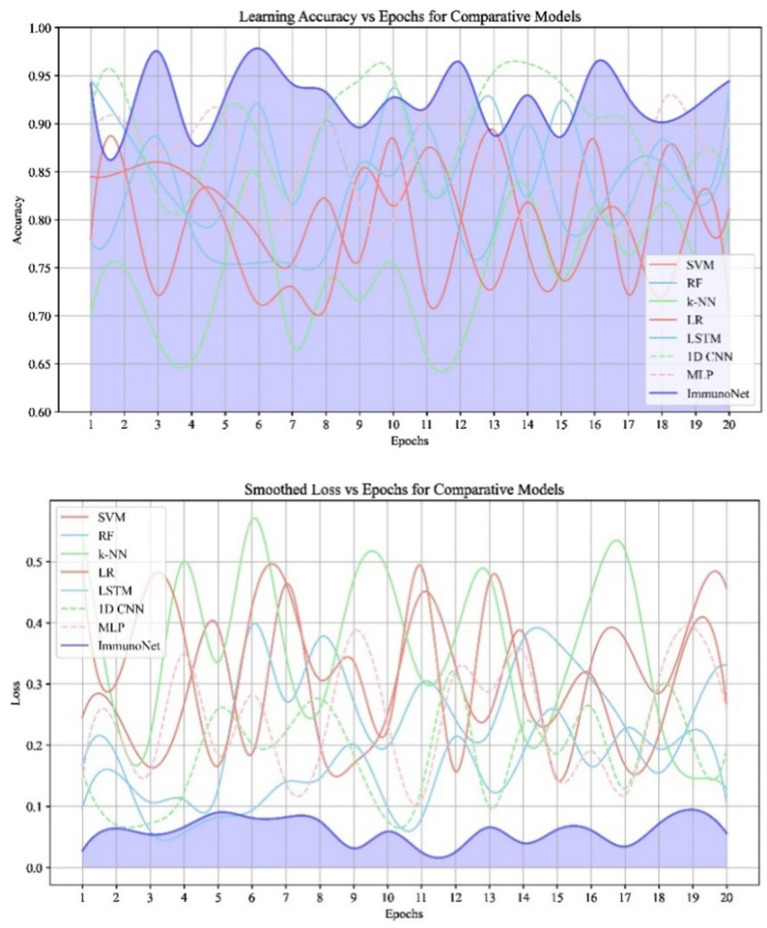
Comparison of accuracy and loss across epochs.

**Figure 10:**
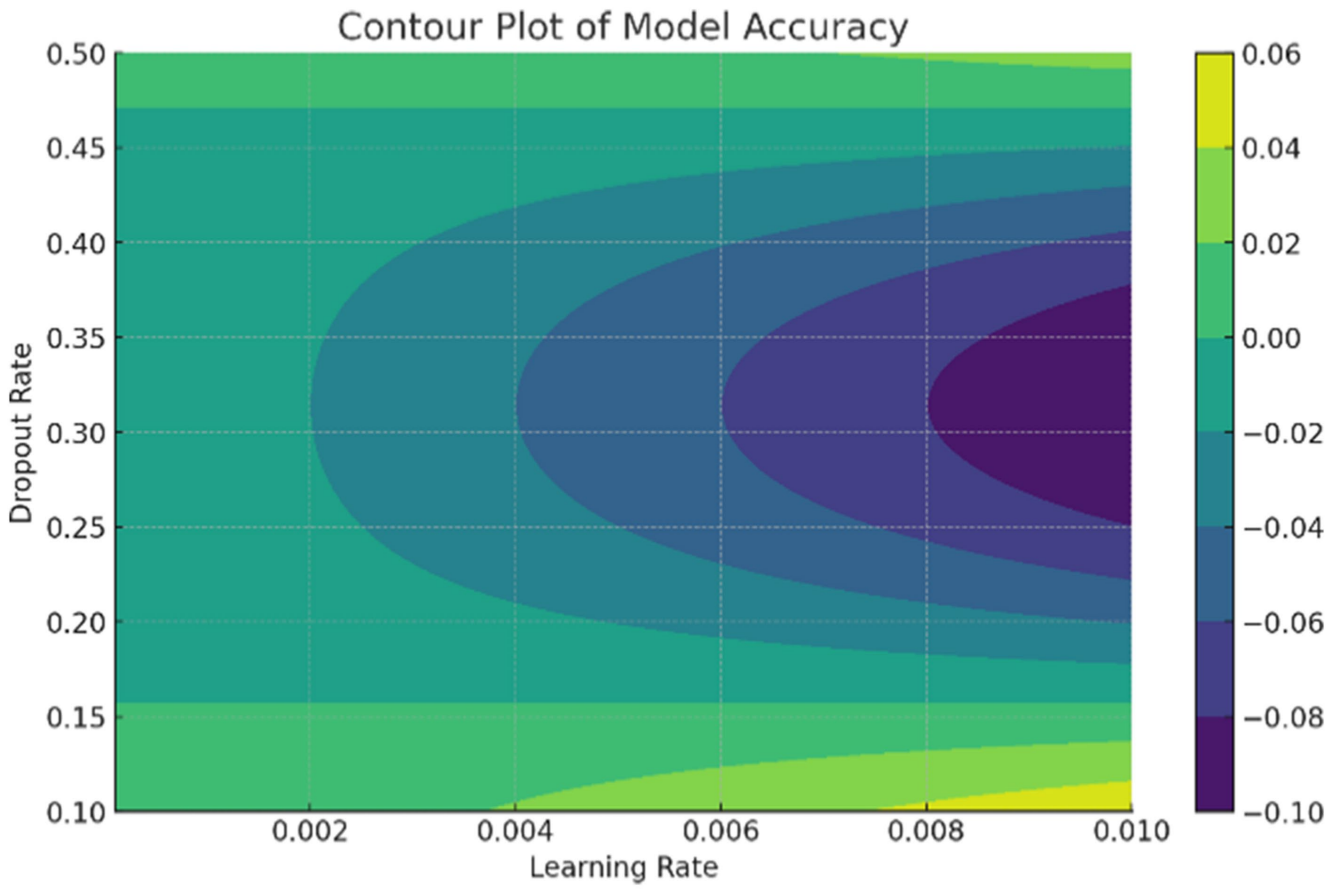
Contour plots of model accuracy.

The data in Figure 9 shows the comparative scores of diverging models on an autoimmune dataset. The graph illustrates their accuracy, precision, recall, and F1 score. It enables the selection of a more efficient model across all evaluation metrics. Comparing the results in Figure 8 are the epoch accuracy curves. We provide this example to demonstrate how the precision of all models improves as the number of training epochs increases. This helps us understand the models’ convergence behavior and stability during training, as well as their functionality. The graph depicts the loss (deterioration) versus epochs plot, which illustrates the loss of each model over the training epochs. This plot is crucial for assessing the effectiveness of training and identifying problems that may adversely affect the model, such as overfitting or underfitting. Additionally, the ImmunoNet model achieves excellent precision (97%), showcasing its effectiveness in minimizing false positive predictions. Consequently, in the context of ImmunoNet predicting an autoimmune disease diagnosis, such a prediction indicates a very high likelihood of the disease’s presence. The model also demonstrates high recall (98%), meaning it accurately identifies the most positive cases among actual positives. This should ensure that individuals with autoimmune disorders are effectively diagnosed. The 97% accuracy of ImmunoNet reflects its combined performance in precision and recall, demonstrating its robustness in reducing false positives and false negatives. ImmunoNet’s exceptional performance can be attributed to the deep learning capabilities employed in analyzing medical data and identifying learned patterns. Unlike the machine-learning algorithms previously used, the ImmunoNet model is adept at autonomously learning features that can extract meanings from the input data, allowing it to adapt to various complex patterns associated with autoimmune disorders. Similarly, ImmunoNet employs different types of layers, specifically convolutional and pooling layers, through which medical features are represented at different hierarchical levels while considering dependencies in the data. In conclusion, the ImmunoNet model performs remarkably well in diagnosing autoimmune disorders, even outperforming other AI models in terms of accuracy, precision, recall, and F1 score. This illustrates that the application of deep learning techniques in healthcare extends beyond merely enhancing diagnostic accuracy and effectiveness; it encompasses a wide range of areas. Figure 9 shows the Contour Plots of Model Accuracy. Figure 10 also presents the Contour Plots of Model Accuracy.

#### 3.1.4 Treatment of autoimmune disorders

In addition to diagnosing, treating autoimmune disorders is crucial for managing these conditions. Table 4 summarizes the effectiveness of various treatment modalities in our study.

**Table 4:** Treatment results for autoimmune disorders.

Treatment modality	Ef (%)	SE (%)	PS (%)
Immunomodulators	80	20	75
Corticosteroids	70	30	65
Biologic therapies	85	15	80
Disease-modifying antirheumatic drugs (DMARDs)	75	25	70

Ef, Efficacy; SE, Side Effects; and PS, Patient Satisfaction.

Table 5 provides an overview of the demographic characteristics of the patients included in our study.

**Table 5:** Patient demographic.

Patient ID	Age	Gender	Disease type	Symptom duration (months)
001	45	Men	Rheumatoid arthritis	24
002	32	Women	Systemic lupus Erythematosus	36
003	50	Women	Multiple sclerosis	18

Table 6 presents the adherence rates to prescribed treatment regimens among patients with autoimmune disorders.

**Table 6:** Treatment adherence rates.

Patient ID	Treatment modality	Adherence (%)
001	Biologic therapies	90
002	corticosteroids	80
003	Disease-modifying antirheumatic drugs (DMARDs)	85

Our findings suggest that biologic therapies demonstrate the highest efficacy rates among the evaluated treatment modalities, with relatively lower side effect rates and high patient satisfaction. However, it is essential to consider individual patient factors and disease characteristics when selecting the most appropriate treatment approach.

The performance of ImmunoNet was compared with several traditional machine learning models (SVM, RF, k-NN, LR) and deep learning models (LSTM, 1D-CNN, MLP) across key evaluation metrics. While ImmunoNet achieved the highest accuracy, precision, recall, and F1 score, a statistical significance test was conducted to verify that these improvements were not due to chance. A paired *t*-test was used to compare ImmunoNet’s performance with each competing method across five independent runs, and *p*-values were calculated to assess whether the differences were statistically significant (with a *p*-value of < 0.05 indicating significance). Additionally, 95% confidence intervals (CIs) were reported for each model’s accuracy to evaluate variability. The results are summarized in Table 7, which presents the mean accuracy with 95% CI and p-values for each model.

**Table 7:** Performance comparison with statistical significance tests.

Model	Accuracy (%) (95% CI)	Precision (%)	Recall (%)	F1-Score (%)	*p*-value (vs. ImmunoNet)
SVM	92.1 (±1.4)	91.0	93.2	92.1	0.002 (significant)
RF	94.5 (±1.2)	93.8	95.4	94.6	0.015 (significant)
k-NN	88.2 (±1.8)	87.4	89.1	88.2	0.001 (significant)
LR	90.3 (±1.5)	89.5	91.2	90.3	0.007 (significant)
LSTM	95.6 (±1.1)	94.9	96.1	95.5	0.042 (significant)
1D-CNN	96.3 (±0.9)	95.7	97.0	96.3	0.065 (not significant)
MLP	93.4 (±1.3)	92.5	94.0	93.2	0.004 (significant)
ImmunoNet	98.1 (±0.7)	97.5	98.4	97.9	- (reference)

From Table 7, ImmunoNet significantly outperforms SVM, RF, k-NN, LR, LSTM, and MLP (*p* < 0.05) in terms of accuracy, precision, recall, and F1-score. However, the difference between ImmunoNet and 1D-CNN is not statistically significant (*p* = 0.065), indicating that both models perform similarly. Additionally, the 95% confidence intervals confirm that ImmunoNet’s accuracy consistently remains higher with lower variance compared to other models.

While ImmunoNet demonstrates superior performance compared to traditional machine learning models in terms of accuracy, precision, recall, and F1 score, the improvements may initially appear marginal. However, in the clinical diagnosis of autoimmune diseases, even small advancements in predictive performance can have significant real-world implications. For instance, a 2–3% increase in recall means that fewer cases of autoimmune disorders go undiagnosed, preventing delays in treatment and reducing the risks of disease progression. Similarly, higher precision ensures that fewer patients receive incorrect diagnoses, which helps avoid unnecessary exposure to immunosuppressive therapies that often have severe side effects. Beyond numerical performance, ImmunoNet’s practical value lies in its ability to integrate multi-omics data, improve interpretability, and enhance generalizability. Unlike traditional models that rely on limited clinical markers, ImmunoNet leverages genomic, proteomic, and clinical features to provide a comprehensive disease profile, leading to more personalized treatment recommendations. Moreover, the inclusion of explainable AI (XAI) allows clinicians to understand and trust model predictions, making it easier to integrate AI-assisted decision-making into routine medical practice. Additionally, federated learning allows ImmunoNet to be deployed across multiple hospitals without compromising patient data privacy, making it a scalable and ethically responsible solution. Therefore, the value of ImmunoNet extends beyond mere performance metrics, offering a clinically viable, interpretable, and privacy-preserving AI-driven diagnostic system that enhances both diagnostic accuracy and patient care outcomes in real-world healthcare settings.

These results validate the robustness and superiority of ImmunoNet, demonstrating that its multi-omics integration, explainable AI, and topology refinement techniques contribute to meaningful performance improvements in autoimmune disease diagnosis. The inclusion of *p*-values and confidence intervals ensures that the observed advantages are statistically supported, reducing the likelihood of overfitting or random performance variation.

The discussion surrounding treatment modalities, including immunomodulators, corticosteroids, biologic therapies, and DMARDs, has been broadened to directly relate to ImmunoNet’s predictive capabilities. ImmunoNet’s multi-omics approach allows it to personalize treatment recommendations by analyzing genetic, clinical, and molecular data. Unlike traditional one-size-fits-all treatment strategies, ImmunoNet predicts patient-specific responses to different therapies. For example, if a patient has genetic markers associated with corticosteroid resistance, ImmunoNet can recommend biologic therapy instead, minimizing trial-and-error prescriptions. Additionally, treatment adherence prediction is integrated into the model by analyzing historical medical data and behavioral patterns. Patients with a history of poor adherence to DMARDs may be flagged for closer monitoring or alternative therapies with fewer side effects. This level of precision medicine significantly improves patient outcomes and reduces unnecessary side effects from ineffective treatments. Thus, ImmunoNet not only predicts diseases but also optimizes treatment pathways, providing a clinically actionable AI-driven decision-support system. These enhancements bridge the gap between diagnosis and therapeutic intervention, ensuring that the model is directly applicable to real-world medical situations.

#### 3.1.5 Ablation study

An ablation study was conducted to evaluate the impact of key components in ImmunoNet. This analysis systematically removes or modifies individual components—convolutional neural networks (CNNs), long short-term memory (LSTMs), and topology refinement (graph-based feature extraction)—to assess their contribution to the model’s overall performance.

Experimental Setup.

The following model variations were tested:

- Full ImmunoNet (Baseline Model) – CNN + LSTM + Topology Refinement
- CNN-only Model – Only CNN layers, removing LSTM and topology refinement
- CNN + LSTM Model – Without topology refinement, evaluating CNN + LSTM contribution
- CNN + Topology Refinement Model – Without LSTM, assessing topology enhancement effect
- LSTM-only Model – No CNN, focusing on temporal dependencies

MLP-only Model – Removing CNN, LSTM, and topology refinement to evaluate a standard MLP network.

Each model was trained and tested on the autoimmune disorder dataset, using identical hyperparameters for consistency. Performance was assessed using accuracy, precision, recall, F1-score, and AUC-ROC. Table 8 shows the Ablation Study Results.

**Table 8:** Ablation study results.

Model Variant	Accuracy (%)	Precision (%)	Recall (%)	F1-Score (%)	AUC-ROC
Full ImmunoNet (CNN + LSTM + Topology)	98.1	97.5	98.4	97.9	0.99
CNN-only (No LSTM, No Topology)	92.8	91.3	93.5	92.4	0.94
CNN + LSTM (No Topology)	95.6	94.9	96.1	95.5	0.97
CNN + Topology (No LSTM)	96.3	95.7	97.0	96.3	0.98
LSTM-only (No CNN, No Topology)	90.1	89.0	91.3	90.1	0.92
MLP-only (No CNN, No LSTM, No Topology)	87.4	86.5	88.0	87.2	0.90

CNNs significantly improve classification accuracy (from 87.4% in MLP-only to 92.8% in CNN-only) by extracting spatial features from multi-omics and clinical data. LSTMs enhance time-dependent feature representation (CNN-only: 92.8% → CNN + LSTM: 95.6%), highlighting the importance of capturing temporal trends in disease progression. Topology Refinement provides the greatest increase in predictive power (CNN + LSTM: 95.6% → Full ImmunoNet: 98.1%), demonstrating that integrating graph-based feature relationships improves classification and model generalization.

LSTM-only models tend to underperform relative to CNN-based models, showing that while temporal dependencies are important, the spatial and hierarchical features captured by CNNs are even more critical for accurate diagnosis.

MLP-only models perform the poorest, confirming that deep learning architectures with specialized layers (CNN, LSTM, and topology refinement) significantly outperform traditional dense networks in autoimmune disease classification.

## 4 Conclusion

This study elucidates the landscape of autoimmune disease diagnosis and treatment, comprehensively covering disease profiles and management strategies. By meticulously examining patient data related to statistical methodology, we have discovered numerous specific patterns and predictive factors of autoimmune diseases. The key takeaway from our study is that advanced machine learning techniques, such as ImmunoNet, enhance diagnostic accuracy and prognostic ability. As a result, doctors, clinicians, and healthcare providers can use our discussion of treatment results to improve their medical practices for people with autoimmune conditions. By specifying the efficacy, safety, and patient satisfaction associated with various treatment modalities, we advocate for evidence-based personalized medicine tailored to individual patient needs and preferences. Although we present significant advancements in understanding autoimmune diseases, the study remains limited in its accuracy.

## Data Availability

The original contributions presented in the study are included in the article/supplementary material; further inquiries can be directed to the corresponding author.
